# *Cordyceps militaris* Exerts Anticancer Effect on Non–Small Cell Lung Cancer by Inhibiting Hedgehog Signaling via Suppression of TCTN3

**DOI:** 10.1177/1534735420923756

**Published:** 2020-05-26

**Authors:** Eunbi Jo, Hyun-Jin Jang, Lei Shen, Kyeong Eun Yang, Min Su Jang, Yang Hoon Huh, Hwa-Seung Yoo, Junsoo Park, Ik Soon Jang, Soo Jung Park

**Affiliations:** 1 Korea Basic Science Institute, Daejeon, Republic of Korea; 2 Hanyang University, Seoul, Republic of Korea; 3 Sungkyunkwan University, Suwon, Republic of Korea; 4 Wonkwang University, Iksan, Republic of Korea; 5 Yonsei University, Wonju, Republic of Korea; 6 Korea Basic Science Institute, Cheongju, Republic of Korea; 7 Daejeon University, Daejeon, Korea; 8 University of Science and Technology, Daejeon, Republic of Korea; 9 Woosuk University, Wanju, Republic of Korea

**Keywords:** apoptosis, TCTN3, GLI1, hedgehog signaling pathway, *Cordyceps militaris*

## Abstract

This study aimed to investigate the effect of *Cordyceps militaris* extract on the proliferation and apoptosis of non–small cell lung cancer (NSCLC) cells and determine the underlying mechanisms. We performed a CCK-8 assay to detect cell proliferation, detection of morphological changes through transmission electron microscopy (TEM), annexin V–FITC/PI double staining to analyze apoptosis, and immunoblotting to measure the protein expression of apoptosis and hedgehog signaling–related proteins, with *C militaris* treated NSCLC cells. In this study, we first found that *C militaris* reduced the viability and induced morphological disruption in NSCLC cells. The gene expression profiles indicated a reprogramming pattern of genes and transcription factors associated with the action of TCTN3 on NSCLC cells. We also confirmed that the *C militaris*–induced inhibition of TCTN3 expression affected the hedgehog signaling pathway. Immunoblotting indicated that *C militaris*–mediated TCTN3 downregulation induced apoptosis in NSCLC cells, involved in the serial activation of caspases. Moreover, we demonstrated that the *C militaris* negatively modulated GLI1 transcriptional activity by suppressing SMO/PTCH1 signaling, which affects the intrinsic apoptotic pathway. When hedgehog binds to the PTCH1, SMO dissociates from PTCH1 inhibition at cilia. As a result, the active GLI1 translocates to the nucleus. *C militaris* clearly suppressed GLI1 nuclear translocation, leading to Bcl-2 and Bcl-xL down-regulation. These results suggested that *C militaris* induced NSCLC cell apoptosis, possibly through the downregulation of SMO/PTCH1 signaling and GLI1 activation via inhibition of TCTN3. Taken together, our findings provide new insights into the treatment of NSCLC using *C militaris*.

## Introduction

Lung cancer is the most commonly occurring life-threatening cancer worldwide.^1^ Non–small cell lung carcinoma (NSCLC) is any type of epithelial lung cancer other than small cell lung carcinoma (SCLC). Currently, NSCLC, the most common subtype (85% of lung cancers), has an overall 5-year survival rate of 16%, which has not significantly improved for several decades.^2^ The poor prognosis of lung cancer can be attributed to the diagnosis of the disease at an advanced stage (only 15% of cases are diagnosed at early stages),^2^ the lack of a cure, as well as the very short survival in patients with advanced stages of NSCLC.^3^ Because NSCLCs are relatively insensitive to chemotherapy and complex, compared with small cell carcinoma, effective long-term therapy is lacking, and hence, there is a need to better understand the biology of lung carcinogenesis. Therefore, understanding the biology of NSCLC carcinogenesis could be crucial for the development of effective therapies.

Cell signaling plays a key role in the development of all multicellular organisms, and many of the proteins currently under investigation as possible targets for cancer therapy are signaling proteins that are components of these pathways.^4^ Especially, the variety of tumor microenvironmental factors causes the chemoresistance of cancer cells; intercellular interactions can also enhance the resistance to anticancer drugs. Therefore, based on the identification of signaling pathways and biomolecular factors of tumorigenesis, various anticancer drugs may be developed for reversing chemoresistance.^5^

Cancer cells have long been known to express embryonic antigens and, in several studies, they have been shown to recapitulate developmental signaling pathways. The hedgehog (HH) signaling pathway, for instance, regulates morphogenesis of various organs during embryogenesis.^6,7^ The HH pathway contains several key components in mammals, including HH glycoproteins, sonic hedgehog (SHH), Indian hedgehog (IHH), and desert hedgehog (DHH).^6^ SHH glycoproteins bind and inactivate Patched1 (PTCH1), which is the 12-transmembrane protein, and normally suppress the activity of Smoothened (SMO), a 7-pass transmembrane-spanning protein, which is a member of the G-protein-coupled receptor superfamily. In the presence of the SHH ligand, PTCH1 inhibition of SMO at the primary cilium is abolished, resulting in nuclear translocation of glioma-associated (GLI) transcription factors, which activate expression of HH target genes, including GLI1 and PTCH genes.^6-8^ The GLI family proteins including GLI1, GLI2, and GLI3 have important roles in the intracellular signaling cascade, and they act as the terminal effectors of the HH signaling.^9^ Notably, GLI1 and GLI2, as glioma-associated oncogenes in the HH signaling pathway, regulate the transcription of multiple downstream target genes and promote tumor progression.^10,11^ Furthermore, dysregulation of the HH pathway has been implicated in several developmental syndromes and cancers.^12,13^ It is already known that the SHH pathway is important for the evolution of radio- and chemoresistance in several types of tumors.^14^ In addition, several studies have reported that HH signaling is activated by the autocrine pathway in NSCLC cells. The aggressiveness of NSCLC has been suggested to be associated with the acquisition of epithelial-to-mesenchymal transition (EMT).^15^ In other research, A549 lung adenocarcinoma cells that obtain mesenchymal phenotype show upregulated SHH and GLI1 expression compared with A549 cells. In the mesenchymal phenotype of A549 cells, the HH pathway was activated by autocrine signaling, and suppression of the HH pathway induced suppression of transforming growth factor-β (TGF-β) signaling–induced cancer cell migration and metastasis.^16^ Also, interesting findings about inhibitors of the HH pathway have renewed hope that disruption of developmental signaling in tumors can be of therapeutic benefit. HH inhibitors block both intrinsic signaling in cancer cells as well as extrinsic signaling to stromal cells to reduce tumor growth.^17,18^ Therefore, tumorigenesis, tumor progression, and therapeutic responses have been shown to be affected by the SHH signaling pathway.^7^ Taken together, HH pathways have been identified as key players in human cancers including NSCLCs.

*Cordyceps militaris* is a genus of parasitic fungi. Traditionally, it has been used as an herbal medicine in Korea and China, to enhance longevity and vitality.^19,20^ A couple of well-known active ingredients in these mushrooms include cordycepin, cordycepic acid, sterols (ergosterol), nucleosides, and polysaccharides.^21^ *C militaris* has been reported to exert immunomodulatory, anti-inflammatory, antimicrobial, and antitumor effects. However, the primary pharmacological activity differs depending on the main ingredients of extract.^22,23^ Evidence from both in vivo and in vitro experiments demonstrated antiproliferative and apoptotic activities of the extracts of *C militaris* in human tumor cell lines, including H460, RKO, PC-3, MDA-MB 231, and HepG2 cells. These extracts exhibited antitumor effects mainly through the induction of apoptosis in tumor cells, inhibition of angiogenesis, and the suppression of invasion and metastasis.^24-27^ Several reports over the past few years have shown that cordycepin (3′-deoxyadenosine), a major bioactive component extracted from *C militaris*, is reported to inhibit cell proliferation,^28-30^ induce apoptosis,^31-33^ inhibit platelet aggregation, regulate steroidogenesis, and reduce inflammation.^34^ Moreover, cordycepin possesses antitumor activities.^35^ In our previous study, we found that cordycepin promotes apoptosis and inhibits proliferation in human ovarian, renal, and lung cancer cells.^36-41^ Also, we investigated the anticancer effect of *C militaris* on human ovarian cancer and renal carcinoma cells. *C militaris* reduced the viability and migration activities, indicative of its potential ability to mediate apoptosis. In addition, apoptosis was induced in human ovarian cancer and renal carcinoma in vitro and in vivo by *C militaris*, which was related to cordycepin.^42,43^ Currently, *C militaris* has received considerable attention worldwide as a potential source of anticancer drugs.^44^ However, the molecular mechanism underlying the *C militaris*–induced inhibition of tumor cell growth and apoptosis remains unclear.

In this study, we attempted to elucidate the apoptotic pathway for *C militaris* to suppress mediated SMO/PTCH1/GLI signaling pathway, thus inducing apoptosis in NSCLC cells. The data presented here clearly showed that *C militaris* is involved in inhibition of the HH signaling pathway and the consequent activation of the caspase family–mediated pathway. Finally, we demonstrated that *C militaris* prevented GLI1 transcriptional activity by suppressing the SMO/PTCH/GLI signaling pathway, and the subsequent activation of intrinsic apoptotic processes induced cancer cell death.

## Materials and Methods

### Preparation of *Cordyceps militaris* Extract

*Cordyceps militaris* was obtained from Wonkwang University, Jeonju Korean Medicine Hospital (Jeollabuk-do, Republic of Korea). Fresh bodies or mycelia of *C militaris* were extracted with 50% ethanol at 80°C for 3 hours (5 times). The *C militaris* extract was filtered using 1-µm pore-size filters, concentrated, and dried. The total extract (200 g, yield [w/w], 11%) was diluted in water.

### Reagents and Chemicals

Fetal bovine serum (FBS) and antibiotic-antimycotic (100×) were procured from Gibco (Waltham, MA), and phosphate-buffered saline (PBS) and F-12 Nutrient Mixture Ham (Ham’s F-12) were purchased from WELGENE Inc (Daegu, Korea). Annexin V-fluorescein isothiocyanate (FITC) Apoptosis Detection Kit was obtained from Sigma-Aldrich (St. Louis, MO). Whole-cell lysis buffer was procured from iNtRON Biotechnology Inc (Seoul, Korea). Antibodies against B-cell lymphoma Bak, Bcl-2, Bcl-xL, caspase-3, and caspase-9 were supplied by Cell Signaling Technology (Beverly, MA), and those against GLI2 and β-actin were obtained from Santa Cruz (Dallas, TX). GLI1, PTCH-1, and SMO antibodies used for immunocytochemistry were purchased from Abcam (Cambridge, UK).

### Cell Lines and Cytotoxicity

The NSCLC cell line A549 (ATCC no. CCL-185) was purchased from the American Type Culture Collection (Rockville, MD), and cultivated in Ham’s F-12 supplemented with 10% (v/v) FBS and 1% (w/v) antibiotic-antimycotic, in a humidified incubator with 5% (v/v) CO_2_ at 37°C. The cells were allowed to adhere and grow for 24 hours prior to the exposure to *C militaris* extract. In brief, A549 cells were seeded in 96-well plates, at a density of 5 × 10^3^ cells/well. After 24 hours of incubation, the cells were treated with various concentrations of *C militaris* extract for 24, 48, and 72 hours. The optimal dose (half maximal inhibitory concentration [IC_50_]) was determined using the cell counting kit (CCK)-8 assay (Dojindo). Briefly, 10 µL of CCK-8 solution was added to each well at the end of the treatment, and the plate was incubated for 2 hours at 37°C. The absorbance was measured at a wavelength 450 nm using a Sunrise microplate absorbance reader (Tecan, Männedorf, Switzerland), relative to that of untreated control in triplicate experiments.

### Apoptosis Analysis by Propidium Iodide (PI)/Annexin V Staining

To determine the apoptotic effects of *C militaris* on the NSCLC cells, we used the annexin V-fluorescein isothiocyanate (FITC) Apoptosis Detection Kit (Sigma-Aldrich). Briefly, the cells were treated with the *C militaris* extract for 48 hours and 72 hours, dissociated using trypsin, and washed twice with PBS. The cell suspension in PBS was centrifuged at 1500 rpm for 5 minutes, and the supernatant was carefully removed by pipetting. The cell pellet was resuspended in 500 µL annexin V binding buffer, and treated with 0.1 µg/mL annexin V-FITC conjugate and 2 µg/mL PI for 10 minutes at room temperature in the dark. The fluorescence of the samples was immediately detected using the Guava system (Millipore) at an excitation wavelength of 488 nm with a 530/30 nm band-pass filter to detect annexin V, and 670 nm high-pass filter to detect PI.

### Transmission Electron Microscopy (TEM)

The *C militaris*–treated NSCLC A549 cells were sequentially fixed with 2.5% glutaraldehyde and 1% osmium tetroxide on ice for 2 hours, and washed with PBS. The tissues were dehydrated in an ethanol and propylene oxide series, embedded in Epon 812 mixture, and polymerized in an oven at 70°C for 24 hours. The sections acquired from the polymerized blocks were collected on grids, counterstained with uranyl acetate and lead citrate, and examined with Bio-HVEM system (JEM-1400Plus at 120 kV and JEM-1000BEF at 1000 kV, JEOL, Japan).

### Microarray Analysis

Transcriptional profiling of the *C militaris*–treated NSCLC was carried out using a human twin 44K cDNA chip. Total RNA was extracted from vehicle- or *C militaris* extract (500 μg/mL)–treated A549, non–small lung cancer cells, and 50 mg RNA was subjected to cDNA synthesis in the presence of aminoallyl-dUTP by reverse transcription. The cDNA was coupled with Cy3 (vehicle), or Cy5 dye (*C militaris*–treated). The genes were thought to be differentially expressed when the global M and log2 (R/G) values exceeded |1.0| (2-fold), at *P* < .05. The Student’s *t* test was performed to assess the statistical significance among the differentially expressed genes after *C militaris* treatment. To analyze the biological significance of these changes, the array data were categorized into specific gene groups.

### Gene Ontology-Based Network Analysis

To study the biological functions of the regulated genes through the interaction network, we used the STRING database (http://string-db.org/), and examined the biological functions of the differentially regulated genes and proteins according to the ontology-related interaction networks, including apoptosis signaling. Network generation was optimized based on the obtained expression profiles with an aim of producing highly connected networks.

### Immunoblotting

Total cell lysates were prepared after the homogenization of cells in 2 mL of Tris-HCl (20 mM) containing a protease inhibitor cocktail (Roche, Basel, Switzerland). The cell homogenate was placed on ice for 30 minutes before centrifugation (10 minutes, 12 000 rpm, 4°C). The protein content in the supernatant was quantified using the bicinchoninic acid method. Denatured proteins (30 µg) were resolved with 12% sodium dodecyl sulfate polyacrylamide gel electrophoresis, and the separated bands were transferred onto a 0.2μm nitrocellulose membrane in a transfer buffer for 2 hours. The membrane was blocked for 1 hour with 5% (w/v) skimmed milk in Tris-buffered saline with Tween-20 (TTBS), followed by incubation with the appropriately diluted primary antibodies at room temperature for 2 hours, or 4°C overnight. After washing the membrane thrice with TTBS, it was probed with horseradish peroxidase–conjugated goat anti-mouse, or rabbit anti-goat IgG (1:2000 dilution) in TTBS containing 5% (w/v) skimmed milk at room temperature for 1 hour. The membrane was rinsed thrice with 0.1% (v/v) TTBS. An enhanced chemiluminescence system (Thermo Scientific, Waltham, MA) was used to visualize the bands on ChemiDoc MP system (Bio-Rad, Hercules, CA). Densitometric measurement of the bands was performed using ImageJ software. Protein levels were quantitatively analyzed after normalization with β-actin level.

### Immunofluorescence Microscopy

The cells were fixed with 4% formamide for 15 minutes at room temperature for 24 hours after the establishment of an adherent culture. The cell membranes were permeabilized with 0.25% Triton X-100 in PBS for 10 minutes, blocked with TBST containing 1% bovine serum albumin (Sigma-Aldrich) for 30 minutes, and incubated with GLI1 primary antibody (Abcam) for 1 hour. The cells were incubated with Alexa Fluor 488–conjugated anti-mouse secondary antibody (Cell Signaling Technology), for 1 hour in the dark. Following treatment with 4,6-diamidino-2-phenylindole, fluorescence images were obtained under a confocal microscope (Nikon, Japan).

### Statistical Analyses

GraphPad Prism (GraphPad, San Diego, CA) was used to perform the statistical analyses. Data were analyzed by 1-way analysis of variance, followed by the Tukey-Kramer multiple comparisons test. The IC_50_ values were determined by nonlinear curve fitting using 5 data points, and expressed as the mean ± standard deviation (SD).

## Results

### *Cordyceps militaris* Dose- and Time-Dependently Suppressed the NSCLC A549 Cell Growth

**Figure 1.**
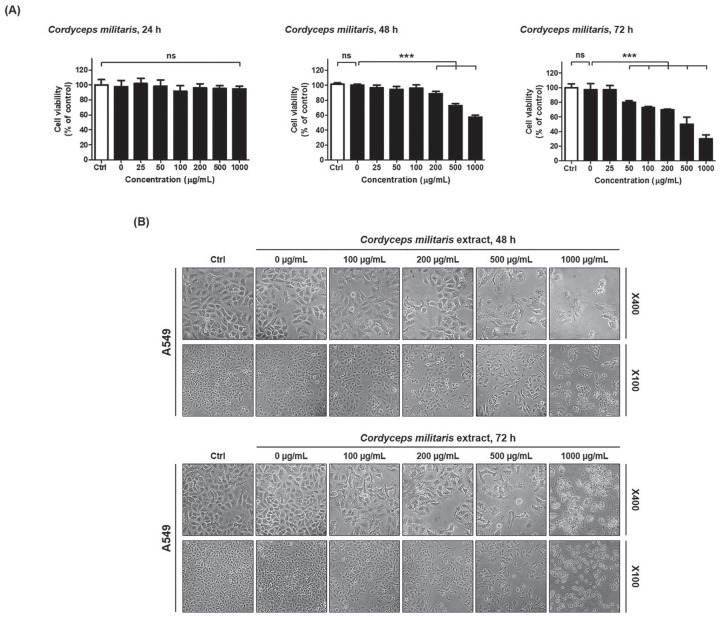
(A) Cell viability after treatment with *Cordyceps militaris* for 24 hours, 48 hours, and 72 hours. (B) Detection of morphological changes by *C militaris* treatment for 48 hours and 72 hours. *Cordyceps militaris* dose- and time-dependently inhibits cell proliferation and induces morphological changes in A549 cells (non–small lung cancer cells). (A) Inhibition of the growth of A549 cells by *C militaris*. A549 were exposed to 0 (vesicle), 25, 50, 100, 200, 500, and 1000 µg/mL *C militaris* extract for 24 hours, 48 hours, and 72 hours prior to estimation of cell number using the CCK-8 assay. The experiment was performed in triplicate. *C militaris* significantly inhibited cell proliferation of A549 cells. (B) Morphological changes of A549 cells treated with *C militaris* compared with control (vehicle). Microscopic images of A549 treated with *C militaris* for 48 hours and 72 hours. Magnification ×100 and ×400. The statistics demonstrated that the percentage of the cells mainly represents treated cells, which was apparent when the percentage of control cells markedly decreased. Data are presented as means ± standard deviations from triplicate experiments. Statistical significance was considered as ***P < .001 versus vehicle treatment; ns, nonsignificance.

To investigate the effects of *C militaris* on NSCLC growth and proliferation, NSCLC cells were treated directly with 0, 25, 50, 100, 200, 500, or 1000 µg/mL for 24 hours, 48 hours, and 72 hours. As shown in Figure 1A, *C militaris* inhibited cell growth during the 24-hour, 48-hour, and 72-hour incubation, in a dose- and time-dependent manner. Treatment with 500 µg/mL *C militaris* extract for 72 hours inhibited approximately half of NSCLC cell populations. Thus, the IC_50_ was determined as 500 µg/mL *C militaris* extract at 72 hours (Figure 1A). To observe the death of *C militaris*–treated cancer cells, the morphologies of NSCLC cells were compared with those of untreated control cells, using light microscopy. The morphology of NSCLC cells changed drastically after treatment with 100 µg/mL *C militaris* extracts for 48 hours (Figure 1B). In contrast, treatment with *C militaris* extract for 24 hours in NSCLC cells had a nonsignificant effect on cell viability (Figure 1A).

Multiple cells began to detach from the surface of the culture plate and appeared buoyant. Moreover, the cells appeared shrunken, resulting in reduced cell volume. These morphological changes preceded apoptosis. On the contrary, 100 µg/mL *C militaris* extracts induced less drastic changes at 48 hours.

### *Cordyceps militaris* Induced Alteration of Apoptotic Gene Expression in the NSCLC Cells

To study the genes involved in the cancer cell growth inhibitory effect of *C militaris*, microarray analysis of *C militaris* (500 µg/mL)–treated A549 NSCLC cells was conducted.

**Figure 2.**
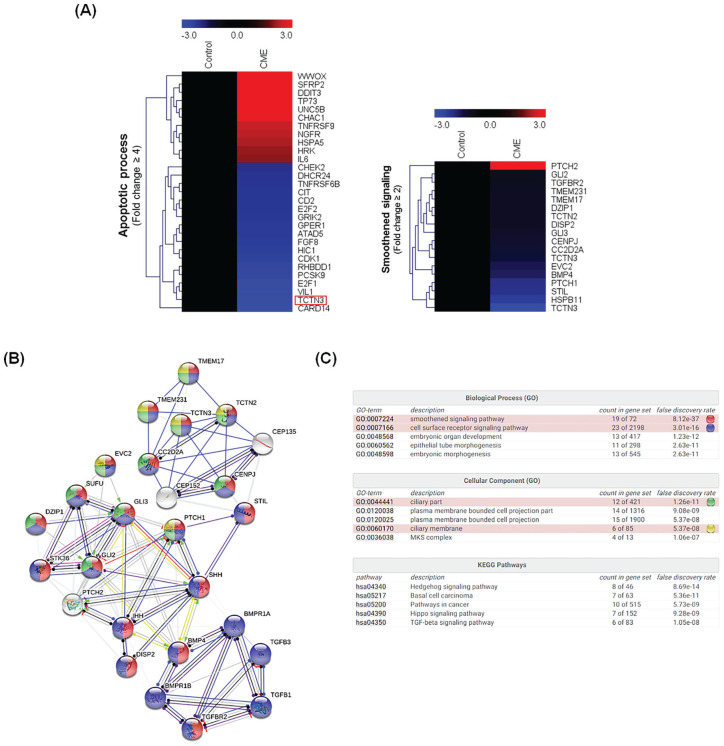
(A) Heap-map clustering analysis of gene expression pattern alternation in A549 treated with *Cordyceps. militaris*. (B) Protein-protein interaction network analysis of gene expression in *C militaris*–treated A549 via STRING v11 (Input genes: Smoothened signaling related genes including PTCH2, GLI2, TGFBR2, TMEM231, TMEM17, DZIP1, TCTN2, DISP2, GLI3, CENPJ, CC2D2A, TCTN3, EVC2, BMP4, PTCH1, STIL, HSPB11, TCTN3). (C) Biological processes, cellular component, and KEGG (Kyoto Encyclopedia of Genes and Genomes) pathway analysis about protein–protein interaction network analysis. Microarray analysis to identify alteration of gene expression and signal network. (A) Hierarchical gene clustering was generated with the TM4 Microarray Software Suite (MeV) from *Cordyceps Militaris* exposed non–small cell lung carcinoma (NSCLC) cells. A heat map revealed genes that were altered more than 2-fold owing to apoptosis (left) and the hedgehog (HH) signaling pathway (right) in response to *C militaris*. The red and blue colors represent more than 3-fold upregulated and downregulated genes, respectively. The ratios of gene profiles are presented as a heat map (left panel), and gene expression pattern (right panel). (B) Combined screenshots from the STRING website, showing results obtained on entering a set of 26 proteins suspected to be involved in the ciliary part and membrane (GO analysis) and HH signaling pathway (KEGG Pathway). The insets show the accessory information available for a single protein, a reported enrichment of functional connections among the set of proteins, and statistical enrichments detected in functional subsystems. (C) Gene Ontology and KEGG Pathway analysis about protein-protein interactions. One enriched function has been selected, and the corresponding protein nodes in the network are automatically highlighted in color.

Among the 58284 genes assayed, 11244 genes were expressed in the *C militaris*–treated cells. Among 8517 genes, *C militaris* treatment upregulated and downregulated 1075 and 7442 genes, respectively, in comparison to the levels observed in the untreated control, at 48 hours. From our gene expression array data, we clustered significantly affected core apoptosis-related genes (Figure 2A). Genes that were upregulated or downregulated more than 2-fold by *C militaris* were categorized as being significant in the data mining. Biologically relevant features were constructed using the Microsoft Excel–based differentially expressed gene analysis (ExDEGA) program. Lists of 4-fold upregulated and downregulated apoptotic process-related genes in *C militaris*–treated A549 NSCLC cells were uploaded to the MeV (Multiple Experiment Viewer) tool for heat maps and hierarchical clusters analysis (Figure 2A). Heat maps and hierarchical clusters demonstrate 29 affected genes in *C militaris* treatment, with 18 genes found to be downregulated and 11 genes upregulated (Figure 2A).

### Protein-Protein Interaction and Gene Ontology Analysis in *Cordyceps militaris*–Treated NSCLC Cells

In Figure 2A, heat maps and hierarchical cluster analysis demonstrated a correlation between *C militaris*–induced apoptosis, and TCTN3 protein expression in *C militaris*–treated NSCLC cells. Tectonic proteins including TCTN1, TCTN2, and TCTN3 are important component proteins residing at the transition zone (TZ) of cilia,^45^ and these are necessary for transduction of the SHH signaling pathway, as revealed by abnormal processing of GLI3 in patient cells.^46^ Therefore, we confirmed whether tectonic and HH signaling pathway–related, including TCTN3, were reduced by *C militaris*. The expression of the tectonic proteins TCTN2 and key proteins of the HH signaling pathway, including PTCH1, GLI2, and GLI3, was reduced in *C militaris*–treated NSCLC cells (Figure 2A, Smoothened signaling, 18 genes). Based on these results, protein-protein interactions and gene ontology analysis, between *C militaris*–induced apoptosis, tectonic proteins, and the HH signaling pathway, were assessed using the STRING database. As a result of pathway analysis, 26 genes including tectonic proteins, and HH signaling pathway–related proteins interacted with each other (Figure 2B). Moreover, pathway analysis comparing non-treated and *C militaris*–treated NSCLC cells revealed that all related proteins were involved in the SMO signaling pathway (GO: 0007224. False discovery rate *P* = 8.12^−37^: 19 genes), cilia part (GO: 0044441. False discovery rate *P* = 1.26^−11^: 12 genes), and ciliary membrane (GO: 0060170. False discovery rate *P* = 5.37^−08^: 6 genes; Figure 2C). In the KEGG analysis, related genes were involved in the HH signaling pathway, basal cell carcinoma pathway, hippo signaling pathway, and TGF-β signaling pathway (Figure 2C). TCTN3 is a protein required for tumorigenesis in association with HH signaling pathway; and thus, we hypothesized that *C militaris*–induced apoptosisin NSCLC cells via inactivation of the HH signaling pathway is caused by inhibition of TCTN3.

### *Cordyceps militaris* Induced Apoptosis in NSCLC Cells

**Figure 3.**
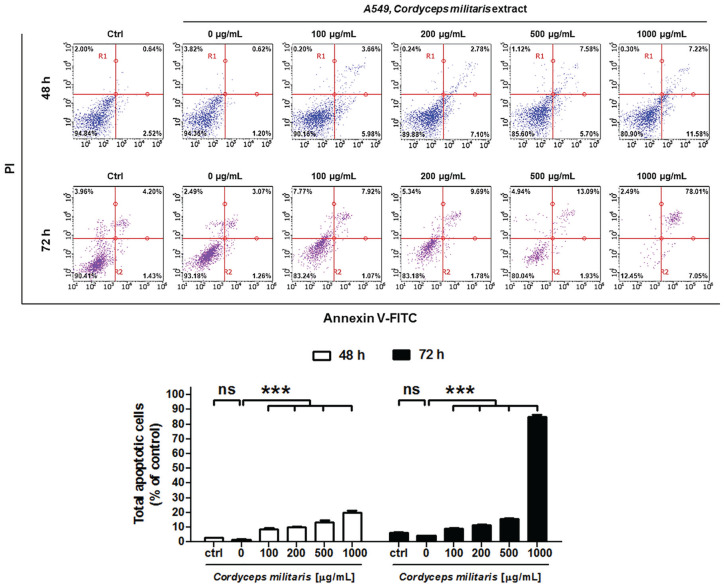
(A) Analysis of apoptosis in *Cordyceps militaris*–treated A549 by Flow Cytometry with classical annexin V/propidium iodide staining. (B) Quantification of total apoptosis induced in *C militaris*–treated A549. *Cordyceps militaris* induces apoptosis in A549 NSCLC cells. Flow cytometry analysis was performed in A549 cells after treatment with the indicated concentrations of *C militaris* for 48 hours and 72 hours. The cells were stained using Annexin V-FITC Apoptosis Detection Kit, and the apoptosis array was determined by the Guava system (Millipore). The statistics showed that the percentage of the cells was mainly represented by early and late apoptosis, which was apparent when the percentage of live cells markedly decreased. Data were normalized to controls and represent the mean ± SD for 3 independent experiments (***P < .001; ns, nonsignificance).

The apoptotic effect of *C militaris* on NSCLC cells was analyzed with annexin V- and PI-stained cells, using flow cytometry after the 48-hour and 72-hour treatment with control, 0 (negative control), 100, 200, 500, and 1000 µg/mL *C militaris*. The assay was performed to evaluate how cancer cell death was induced by *C militaris*. The relative proportion of nonviable cells was quantitatively measured as those at the early stage of apoptosis (annexin V–stained, nondisrupted cells), or as those entering the late stage of apoptosis (disrupted or lysed cells). In 100 µg/mL *C militaris*–treated cells at 48 hours, no drastic change in the annexin V–stained viable fraction was observed (94% to 90%; Figure 3). However, the NSCLC cells treated with 200, 500, and 1000 µg/mL *C militaris* extract for 48 hours markedly shifted from the normal to the apoptotic stage (3.16% to 9.88%, 13.28%, and 18.3%), and the viable fraction at 1000 µg/mL was reduced from 94% to 80%. Moreover, NSCLC cells treated with 1000 µg/mL *C militaris* extract for 72 hours reported a reduction in the viable fraction from 90.41% to 12.45%, rapidly increasing the apoptotic stage from 5.63% to 85.06%. Therefore, *C militaris* dose- and time-dependently induced the apoptotic process in NSCLC cells (Figure 3).

### *Cordyceps militaris–*Treated NSCLC Apoptotic Bodies Observed by TEM

**Figure 4.**
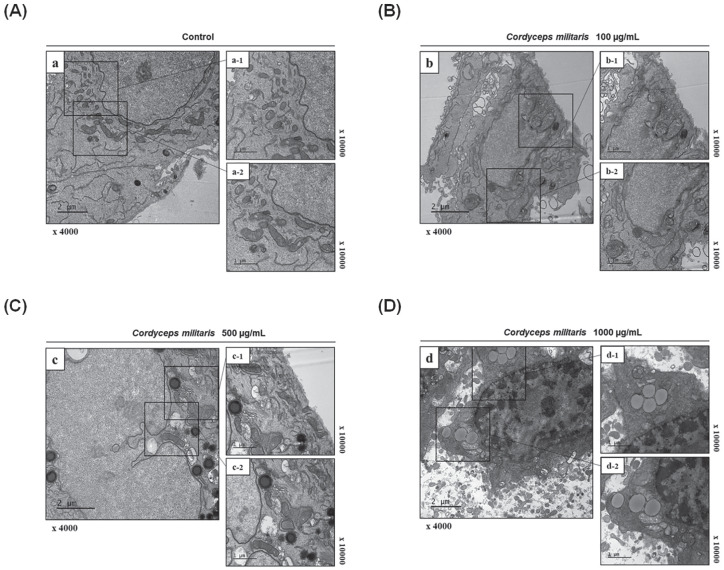
Morphological ultrastructural appearance of apoptotic bodies by transmission electron microscopy (TEM). Morphological ultrastructural appearance of apoptotic bodies by transmission electron microscopy (TEM) in *Cordyceps militaris–*treated non–small cell lung carcinoma (NSCLC) cells. (A) Untreated A549 and A549 were incubated with *C militaris* at (B) 100 µg/mL, (C) 500 µg/mL, and (D) 1000 µg/mL for 48 hours and analyzed by TEM. The typical apoptotic bodies in *C militaris*–treated A549 cells were spherical protuberances containing fragmentation, and segregation of chromatin clumps separated from cell surface. Mitochondrial disruptions, autophagosomes, and autolysosome were detected in *C militaris–*exposed A549 cells. Representative images are shown.

To further confirm the development of apoptotic bodies following *C militaris* treatment, and to better visualize the ultrastructural changes occurring in A549 apoptotic cells, we used electron microscopy (Figure 4). Unlike the control and 100 µg/mL of *C militaris* extract (Figure 4A and B), apoptotic bodies were observed in 500 and 1000 µg/mL *C militaris*–treated cells. These bodies were spherical protuberances containing fragmented and segregated chromatin clumps, separating from the cell surface (Figure 4C and D). *C militaris*–treated cells accumulated damaged mitochondria and autophagosomes, or autolysosome containing dense organelles, 2 days after the treatment (Figure 4C). Conversely, the untreated NSCLC cells had intact plasma membranes and an ordered chromatin damage (Figure 4D).

### *Cordyceps militaris* Extract Increased Apoptotic-Related Proteins in NSCLC Cells

**Figure 5.**
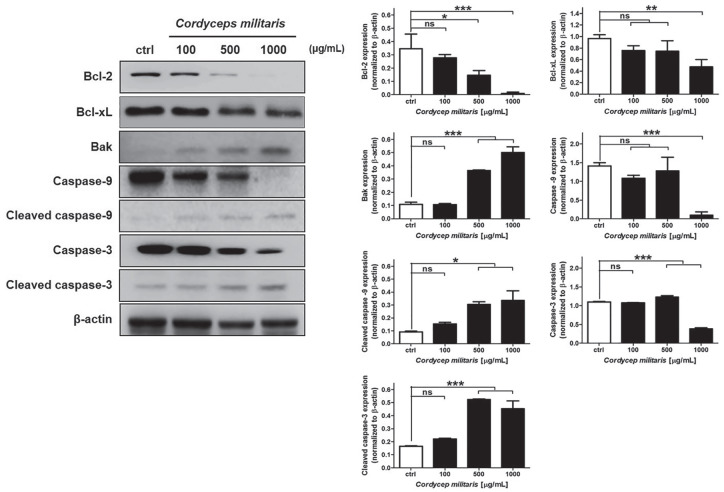
Detection of apoptotic protein expression changes in *Cordyceps militaris*–treated A549 via immunoblotting and quantification of protein expression. *Cordyceps militaris* induces alternation of apoptotic protein expression in non–small cell lung carcinoma (NSCLC) cells. The NSCLC cell lines were exposed to 0, 100, 500, and 1000 µg/mL *C militaris* extract for 48 hours before whole-cell protein lysates were harvested and prepared for western blot analysis using Bak, Bcl-2, Bcl-xL, cleaved caspase-9 and caspase-3. Data were normalized to controls and represent the mean ± SD for 3 independent experiments (*P < .05, **P < .01; ***P < .001; ns, nonsignificance).

To study the mechanism by which *C militaris* inhibits cell proliferation and induces cell apoptosis, NSCLC cells treated with different doses of *C militaris* extract (0, 100, 500, and 1000 µg/mL) were used for protein expression analysis. Bak and Bcl-2, Bcl-xL and proapoptotic members, were analyzed as target proteins using immunoblotting. The results demonstrated that the protein expression levels of the cleaved caspase-3 and caspase-9 were increased significantly after treatment with the *C militaris* extract (Figure 5). Taken together, these results implied *C militaris*–induced cell apoptosis through Bak, Bcl-2, Bcl-xL, and caspase-dependent pathways (Figure 5). TCTN3 has been shown to be a key positive regulator of apoptosis by inhibiting the activation of caspases in the NSCLC cells, via the suppression of the HH signaling pathway. Therefore, we additionally evaluated whether *C militaris* influenced the HH signaling pathway in NSCLC cells.

### *Cordyceps militaris* Extract Induced Apoptosis via Inhibition of GLI1 Nuclear Translocation by Regulating HH Signaling Pathway in NSCLC Cells

**Figure 6.**
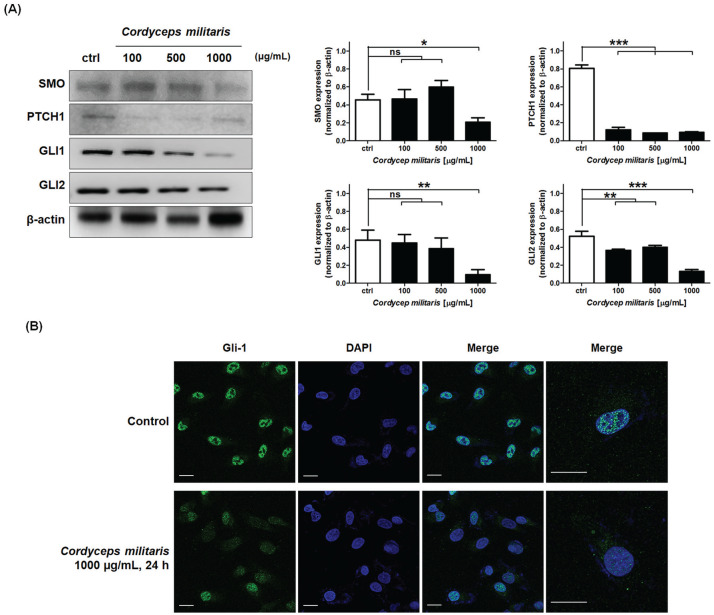
(A) Detection of hedgehog (HH) signaling–related protein expression alternation induced by *Cordyceps militaris* in A549 via immunoblotting and quantification of protein expression. (B) Detection of GLI-1 translocation from cytosol to nucleus induced by *C militaris* in A549 via immunofluorescence analysis. *Cordyceps militaris* suppresses GLI1 transcriptional activity via suppression of SMO/PTCH1 signaling (A) Effect of SMO/PTCH1 signaling on nuclear translocation of GLI1 in *C militari*s A549 cells. (B) Immunofluorescence micrographs of GLI1 translocation in NSCLC cells. *C militaris* decreases nuclear GLI1 level in NSCLC cells. NSCLC cells were incubated with 1000 µg/mL drug or vehicle for 24 hours. They were fixed and probed with GlI-1 antibody. GLI1 translocation into the nucleus was detected with an anti-GLI1 antibody (green fluorescence); bar, 10 µm. The data represent the mean ± SD from 3 independent experiments (*P < .05; **P < .01; ***P < .001; ns, nonsignificance).

Currently, studies are ongoing to evaluate the potential role of HH inhibitors as solid tumor targeted therapies, for example, in NSCLC.^47^ To further investigate whether SMO/PTCH1/GLI1, GLI2 were functionally linked to caspase signaling, we examined the effect SMO/PTCH1 on activation of GLIs. Signaling pathways operative during organ development, including SHH and associated GLI transcription factors (GLI 1-3), have recently been found to be reactivated in NSCLC.^1^ Therefore, we confirmed the alteration of SMO, PTCH1, and GLI1/2 expression by *C militaris* in the NSCLC cells. In addition, the activity of GLI1 was determined by the activation of the SHH pathway, and transcriptional activity of GLI1 was identified. SMO and GLI1/2 expression were downregulated at 1000 µg/mL *C militaris* extract, and PTCH1 expression was decreased at 100 µg/mL of *C militaris* extract (Figure 6A). Next, we investigated the effects of SMO/PTCH1 on GLI1 transcriptional activity, induced through *C militaris* in the NSCLC cells. Thus, to confirm the activation of GLI1, we detected translocation to the nucleus. Since the expression of TNFR2 did not occur in A549, IL-1, not TNF-α, was used to induce the translocation of GLI1. However, *C militaris* extract treatment of A549 decreased the nuclear translocation of GLI1 (Figure 6B). These results indicated that *C militaris* attenuated SMO/PTCH1-mediated GLI1 transcriptional activity, to induce intrinsic apoptosis in NSCLC cells.

## Discussion and Conclusion

Identification of natural products to overcome insensitivity and resistance to anticancer drugs represents an important strategy since drug insensitivity and resistance lead to treatment failure in clinic and remains an obstacle in cancer therapy.^48^ Management of drug insensitivity and resistance is important for successful chemotherapy. There are many reports that natural products can overcome cancer cell drug resistance, which deserve sharing with scientific and industrial communities.^49^ Considering the complexity of various medicinal herbs, application of artificial intelligence technology may promote the development of anticancer drugs from many medicinal herbs.^50,51^

Although *C militaris*–induced cell death has been reported, the molecular mechanisms that mediated the *C militaris–*induced apoptosis remained unknown in NSCLC. Here, we focused on understanding the fundamental mechanisms of *C militaris*–induced apoptosis, and examined the relationship between TCTN3/SMO/PTCH1 expression, and GLI1 transcriptional activity.

*Cordyceps militaris* has been extensively studied with regard to various biological functions, including antiviral, antioxidant, anti-inflammatory, and antitumor activities.^52^ In particular, research has reported the anticancer activity of *C militaris*.^21,27,53^ In this study, we demonstrated the anticancer effect of *C militaris* in A549 NSCLC cells. We found that *C militaris* inhibited the growth and proliferation of NSCLC cells in a dose- and time-dependent manner, verifying its apoptotic potential (Figure 1).

In addition, flow cytometric analysis revealed that *C militaris* increased apoptosis in the NSCLC cells in a dose- and time-dependent manner. Approximately 20% of NSCLC cells exhibited early- and late-phase apoptosis following treatment with the *C militaris* extract (1000 µg/mL) for 48 hours, and 72 hours posttreatment, the NSCLC cells were altered from 90.41% to 12.45%, rapidly increasing the apoptotic stage from 5.63% to 85.06% (Figure 3). Next, using TEM, we visualized the apoptotic bodies of *C militaris*–treated NSCLC cells (Figure 4). Extensive mitochondrial damage and dysfunction was indicated in a dose-dependent manner, especially the autophagosome and autolysosome formation (Figure 4B and D). The untreated control cells showed normal organelles without apoptotic bodies.

Several studies have described the noncanonical activation of the HH pathway, especially in cancers,^54^ and the noncanonical activation of SHH pathway involved the activation of GLI proteins, independent of SHH, PTCH, and SMO.^55^ Especially, HH signaling enhances EMT transition of NSCLC cells, leading to increased proliferation and invasion.^28^ Moreover, this pathway is reportedly involved in tumor drug resistance in lung cancer.^56^

Cordycepin, the major active compound in *C militaris*, induces human lung cancer cell apoptosis by inhibiting the nitric oxide–mediated ERK/Slug signaling pathway,^39^ and inhibits drug-resistant NSCLC progression by activating the AMPK signaling pathway.^57^ These data suggest that *C militaris* is involved in the regulation of apoptosis-related signaling pathways. Using microarray analysis, we examined the *C militaris*–induced apoptotic gene and protein expression pathways in NSCLC cells.

As mentioned above, HH signaling is critical for epithelial mesenchymal transition and invasion in NSCLCs. In addition, HH signaling is also important for migration and invasion in various human cancers. For example, in breast cancer cells, SHH/GLI1 positive tumor demonstrated high expression of Snail and Vimentin with relatively low expression of E-cadherin, and SHH/GLI1 axis inactivation also significantly restricted migration and invasion.^58^ Primary cilia have an essential role in HH signaling in mammals, and these structures are essential for developmental signaling through the HH pathway.^59^ The primary cilium, by recognizing signals from the extracellular environment, and by displaying receptors required for signal interception, as well as the downstream molecular effectors, is a key mediator of the impaired signaling that induces malignancy. Also, the presence or absence of primary cilia can regulate each event that is essential for cancer survival, thus accentuating our inability to define a precise role for the primary cilium in cancer progression.^60^ The transition zone (TZ) is a specialized ciliary domain present at the base of the cilium, and it is part of a gate that controls protein entry and exit from this organelle.^61^ Tectonic proteins (TCTN1, TCTN2, and TCTN3) are important component proteins residing at the TZ of cilia. Indeed, many ciliopathies have been reported to involve tectonic mutations, highlighting a crucial role for tectonic proteins in ciliary functions. More important, tectonic proteins play a vital role in the regulation of the SHH pathway.^45^ Also, tectonic proteins are an upstream gene involved in embryonic development. The aim of the further study is to investigate the effect of the tectonic proteins gene on the viability and migration of human cancer cells. For example, Dai et al reported that knockdown of TCTN1 suppressed cell growth by inducing cell cycle arrest and apoptosis in colorectal cancer and that TCTN1 might act as an underlying oncogene that plays a crucial role in the occurrence and development of colorectal cancer.^62^ Our results indicated that expression of 248 genes was altered by *C militaris*, and heat maps and hierarchical clusters demonstrated 29 affected genes in the lists of 4-fold upregulated and downregulated apoptotic process–related genes (Figure 2A). We also found that apoptosis of A549 induced by *C militaris* was associated with the HH signaling pathway via the reduction of TCTN3 expression (Figure 3). As we mentioned, tectonic proteins play a vital role in the regulation of the HH pathway in primary cilia.^46^ TCTN1, TCTN2, and TCTN3 share obvious similarities in their conserved functions such as neural patterning and GLI3 processing.^63^ Therefore, we analyzed the expression alteration of key proteins related to HH signaling pathway induced by *C militaris*, and confirmed whether they are involved in apoptosis induction of NSCLC cells. However, the effect of *C militaris* on the basic players in the HH signaling pathway remains unknown, and further research is needed to elucidate the detailed molecular mechanism mediated by *C militaris*.

In a previous study conducted by Yuan et al using a tumor tissue array containing 120 NSCLC samples, GLI1 was found to be expressed in the majority of lung adenocarcinoma and squamous cell carcinomas, indicating a basal HH activity in NSCLC cells.^64^ Nonetheless, no assessment of the GLI1 expression was performed in the stromal cancer compartment and no correlation was established with patient follow-up.

Our results showed that *C militaris* decreased the expression of SMO, PTCH1, and GLI1/2 (Figure 6A), and reduced the GLI1 translocation to the nucleus (Figure 6B), increased Bak, cleaved caspase-3, and cleaved caspase-9, and decreased Bcl-2 and Bcl-xL (Figure 5). These findings indicated that the inhibition of TCTN3 suppressed the SMO/PTCH1 signaling pathway, resulting in the inactivation of the GLI1 transcriptional activity by *C militaris* in NSCLC cells.

In summary, our results demonstrate that *C militaris* triggers apoptosis by suppressing the SMO/PTCH1/GLI1 signaling pathway, via inhibition of TCTN3 expression. In addition, *C militaris* promotes the cleavage of caspase-3 and caspase-9 by inducing intrinsic apoptosis in NSCLC cells. Our findings describe the molecular mechanisms of apoptosis induced by *C militaris*, which may provide a theoretical basis for the application of *C militaris* derivatives in cancer therapy.
